# Evaluation of the Naples Prognostic Score in Patients with Head and Neck Squamous Cell Carcinoma

**DOI:** 10.3390/nu18081196

**Published:** 2026-04-10

**Authors:** Magdalena Wissa, Alexander Lein, Bernhard J. Jank, Gregor Heiduschka, Lorenz Kadletz-Wanke, Faris F. Brkic

**Affiliations:** 1 Department of Otolaryngology, Head and Neck Surgery, Medical University of Vienna, 1090 Vienna, Austria; magdalena.wissa@hotmail.com (M.W.); gregor.heiduschka@meduniwien.ac.at (G.H.); faris.brkic@meduniwien.ac.at (F.F.B.); 2 Department of Otorhinolaryngology, University Hospital Wiener Neustadt, 2700 Wiener Neustadt, Austria; bernhard.jank@meduniwien.ac.at (B.J.J.); lorenz.kadletz@meduniwien.ac.at (L.K.-W.)

**Keywords:** head and neck squamous cell carcinoma, prognostic marker, Naples Prognostic Score, biomarker

## Abstract

Background/Objectives: Systemic inflammation and nutritional status are two factors known to influence the prognosis of cancer patients. The Naples Prognostic Score (NPS) includes two inflammatory and two nutritional markers and combines them in a single score to better predict survival of cancer patients. In this study, we aimed to critically evaluate the NPS for its significance in head and neck cancer patients. Methods: We retrospectively analyzed the preoperative NPS and its association with overall survival (OS) and disease-free survival (DFS). We evaluated 140 patients with head and neck squamous cell carcinoma (HNSCC) who were treated with primary surgical therapy at a tertiary center between 2001 and 2019. OS and DFS were analyzed using Kaplan–Meier estimators, the log-rank test and uni- and multivariable Cox models. Results: The median postoperative follow-up was 6.7 years. Higher NPS showed numerically, but not significantly shorter OS and DFS. Sensitivity analysis for all markers included in the NPS revealed only the neutrophil-to-lymphocyte ratio (NLR) as a significant predictor for OS and DFS. Conclusions: These findings suggest that the overall NPS may have limited prognostic value in this cohort. In contrast, NLR appears to be a more robust and clinically relevant marker for survival outcomes in patients with head and neck squamous cell carcinoma.

## 1. Introduction

Head and neck squamous cell carcinoma (HNSCC) account for approximately 90 percent of malignant tumors of the head and neck and represent the seventh most common malignancy. The estimated worldwide incidence is over 830,000 new cases and approximately 430,000 deaths occur per year [1]. Treatment modalities can include surgery, radio- or chemoradiotherapy and antibody-based therapy depending on the disease stage [2]. Despite advantages in all therapeutic fields of HNSCC treatment in recent decades, outcomes have remained mostly unchanged [2].

The best predictor of survival to date is the TNM classification, a system for staging of solid tumors based on the anatomical extent of the tumor. Besides parameters of the TNM classification, namely tumor size, lymph node metastasis status and distant metastasis status, pathological findings like vascular and neural invasion and histopathological grading are also included factors. Notably, the 8th edition of the American Joint Commission on Cancer (AJCC) has included a separate staging for HPV-positive oropharyngeal squamous cell carcinoma (OPSCC) patients. The update acknowledges the significant better prognosis of HPV-associated OPSCC [3], and underlines the incomplete representation of neoplastic diseases by an anatomically based tumor classification alone. HNSCC in general are tumors with a high heterogeneity and therefore can show disparities in treatment response independent of TNM stage [4]. A more detailed characterization of HNSCC patients to better predict the course of the disease and guide treatment decision is therefore urgently needed.

Serum-based biomarkers for systemic inflammation and nutrition are host-factors that have been investigated for their prognostic significance in numerous cancers. Some of those markers can predict disease outcome in a large number of different cancers [5,6]. Notably, those markers can be determined by pre-treatment peripheral blood sampling and are therefore simple, inexpensive and easily accessible in the clinic. However, most of those markers lack a defined cutoff, limiting their generalizability and broad acceptance.

The Naples Prognostic Score (NPS) is a newly proposed scoring system with predefined cutoffs. It includes serum albumin, the most abundant plasma protein, reflecting both nutritional status and systemic inflammatory response, making it a key component of the NPS [7]. The NPS also includes cholesterol to represent the patient’s nutritional status, and neutrophil-to-lymphocyte ratio (NLR) and lymphocyte-to-monocyte ratio (LMR) to represent the patient’s systemic inflammation status. It was first described by Galizia et al. in 2017 in a cohort of colorectal cancer patients [8]. Since then, the NPS has been validated in osteosarcoma [9], endometrial cancer [10], early-stage lung cancer [11], and pancreatic cancer [12] as a prognosticator for disease outcome. Nevertheless, as the NPS integrates parameters reflecting both nutritional and inflammatory status, its components may be influenced by patient- and disease-related factors, which could affect its prognostic performance across different types of carcinomas [8].

The aim of this study was to critically evaluate the NPS and its association with disease outcome in HNSCC patients who underwent curative surgical treatment.

## 2. Materials and Methods

This study is a single-center retrospective cohort study. Patients diagnosed with HNSCC between 2002 and 2018 treated with primary surgical therapy with curative intent were screened for inclusion. Exclusion criteria were prior external treatment, secondary primary carcinoma and immunosuppression. The last follow-up occurred in September 2019. Baseline and outcome data were collected retrospectively from electronic patient records, including the following data: age at surgery, sex, tumor site, HPV status, TNM classification in accordance with the 8th edition of the AJCC, disease-free and overall survival. Smoking status and alcohol consumption were recorded, as both are well-established risk factors in HNSCC and may influence survival outcomes. Furthermore, these factors may influence systemic inflammatory responses and nutritional status, potentially confounding the relationship between NPS and clinical outcomes [13,14,15]. In addition to that, postoperative radio- and/or chemotherapy received, serum albumin, total cholesterol, neutrophil-, lymphocyte- and monocyte count were documented. Serum markers were included between 3 weeks and one day prior to surgery. The complete dataset could be extracted for 140 patients.

The primary outcome for this study was overall survival and the secondary outcome was disease-free survival. Cutoffs for all markers were based on the work by Galizia et al. and were as follows: serum albumin > 4 mg/dL, total cholesterol ≤ 180 mg/dL, NLR > 2.96, LMR ≤ 4.44. If the cutoff was reached, one point was added to the NPS. Subsequently, patients with 0 points were assigned to NPS group 0, patients with 1 or 2 points to NPS group 1 and patients with a NPS of 3 or 4 to group 2 [8]. To analyze the effect of individual markers included in the NPS, we performed a sensitivity analysis. We therefore excluded individual parameters and analyzed its effect on the NPS and its prognostic power for OS and DFS. Continuous variables were summarized as medians with interquartile range (Q1–Q3) and categorial variables were summarized as absolute counts and percentages (%). The median follow-up time was estimated with a reverse Kaplan–Meier estimator according to Schemper et al. [16]. Kaplan–Meier estimator was used to analyze rates for OS and the Kaplan–Meier failure function was used to analyze DFS. Differences between groups were analyzed using the log-rank test. For modeling, we used uni- and multivariable cox proportional hazard models. All multivariable models were adjusted for HPV status, smoker status and stage (dichotomized; stages I–II vs. III–IV). Hazard ratios (HR), 95% confidence interval (95% CI) and *p*-values were calculated. A two-sided *p*-value < 0.05 was considered statistically significant. Statistical analysis was performed using Stata (Macintosh version 14.0, StataCrop LLC, College Station, TX, USA).

## 3. Results

### 3.1. Analysis at Baseline

One hundred forty patients, who had undergone surgery with curative intent for HNSCC, could be included. The baseline characteristics of our cohort are summarized in Table 1. In brief, 30 patients (21%) were female and the median patient age at diagnosis was 60.6 years (25th to 75th percentile: 54–65 years). Tumors were localized at the hypopharynx in 13%, the larynx in 9%, the oral cavity in 21% and the oropharynx in 57%. Fifty-five patients (40%) were HPV-positive, 42 (34%) were active drinkers and 77 (57%) were active smokers. Eight patients received surgical treatment only, 108 (77%) received surgery followed by postoperative radiotherapy and 24 (17%) received surgery followed by postoperative chemoradiotherapy. The median preoperative serum albumin was 4.3 mg/dL (3.9–4.5), total cholesterol was 191 mg/dL (166–225), NLR was 2.78 (2.1–3.8) and LMR was 2.89 (2.29–4.17). Fifteen patients had a calculated Naples score of 0 (11%), 38 of 1 (27%), 49 of 2 (35%), 32 of 3 (23%) and 6 of 4 (4%). After assigning patients to the Naples score group as described previously, 15 patients were assigned to group 0 (11%), 87 patients to group 1 (62%) and 38 patients to group 2 (27%) (Table 1).

### 3.2. Disease Outcome and Its Association with NPS

**Figure 1:**
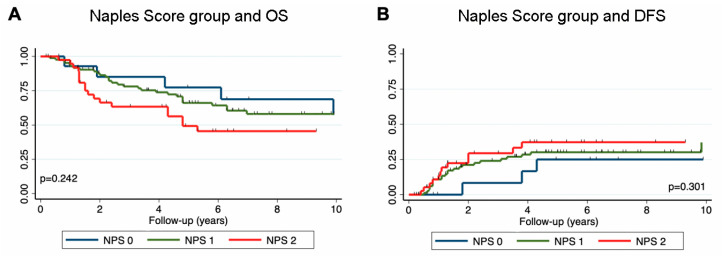
Kaplan–Meier curves for overall survival (**A**) and disease-free survival (**B**) separated by the Naples Prognostic Score group.

Disease recurrence occurred in 42 patients (30%) with a median DFS of 1.3 years (25th–75th percentile: 0.8–3.3 years). Fifty-six patients (40%) died during a median follow-up of 6.7 years. The 5-year overall survival was calculated as 64% (95% CI: 55–71%). In survival analysis using Kaplan–Meier estimators and log-rank test, the Naples score groups showed numerically worse overall survival with increasing group (5-year OS: group 0 = 77.8%; 1 = 67.3%; 2 = 50%); however, this was not statistically significant (*p* = 0.242; Figure 1A). Similarly, an increasing NPS showed a numerically worse DFS (5-year DFS: NPS 0 = 77%; 1 = 68.5%; 2 = 61.3%, *p* = 0.301), this was also not statistically significant (*p* = 0.301, Figure 1B).

In univariable analysis, no associations between the Naples score with OS (HR per 1 increase: 1.275, 95% CI: 0.97–1.67, *p* = 0.079) or DFS (HR per 1 increase: 1.302; 95% CI: 0.96–1.75, *p* = 0.082) could be observed. This result prevailed after correction for possible confounders in multivariable analysis (Table 2).

**Figure 2:**
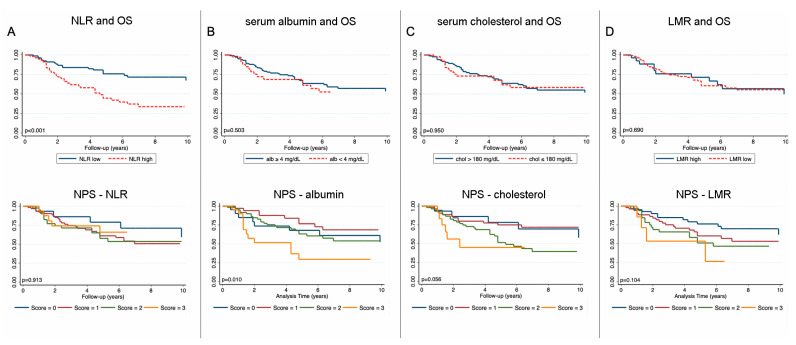
Sensitivity analysis of the NPS for OS. Kaplan–Meier curves for each marker included in the NPS separately (**top row**) and the effect on the NPS after exclusion of each individual marker (**bottom row**). (**A**) NLR in relation to OS and the corresponding NPS after exclusion of NLR; (**B**) serum albumin in relation to OS and the corresponding NPS after exclusion of albumin; (**C**) serum cholesterol in relation to OS and the corresponding NPS after exclusion of cholesterol; (**D**) LMR in relation to OS and the corresponding NPS after exclusion of LMR.

**Figure 3:**
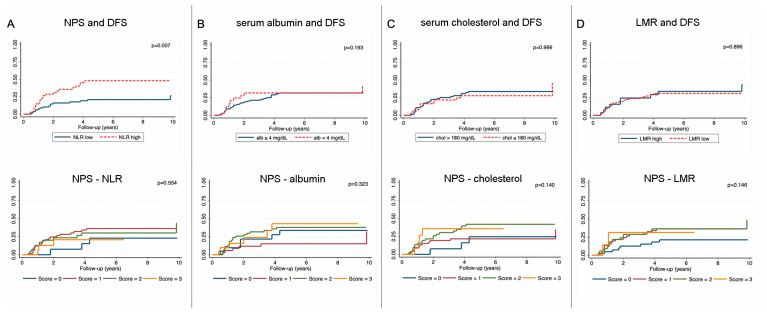
Sensitivity analysis of the NPS for DFS. Kaplan–Meier failure function for each marker included in the NPS separately (**top row**) and the effect on the NPS after exclusion of each individual marker (**bottom row**). (**A**) NLR in relation to DFS and the corresponding NPS after exclusion of NLR; (**B**) serum albumin in relation to DFS and the corresponding NPS after exclusion of albumin; (**C**) serum cholesterol in relation to DFS and the corresponding NPS after exclusion of cholesterol; (**D**) LMR in relation to DFS and the corresponding NPS after exclusion of LMR.

Since we found a numerically worse, but not statistically significant OS and DFS with increasing NPS, we subsequently performed a sensitivity analysis for all markers included in the NPS. A strong association was found for NLR with OS (5-year OS; NLR high vs. low: 76% vs. 47%, *p* =< 0.001, corrected *p* = 0.003, Figure 2A) and DFS (5-year DFS: 78% vs. 51%, *p* = 0.007, corrected *p* = 0.028, Figure 3A). No associations could be found for all other markers with OS or DFS (Figure 2B–D and Figure 3B–D).

**Figure 4:**
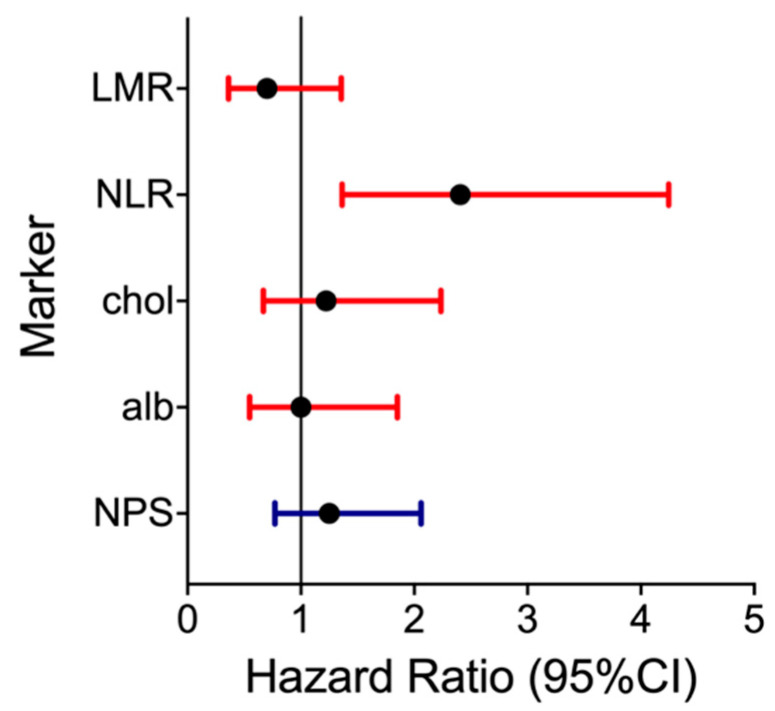
Forrest plot of hazard ratios and 95% CI for overall survival of the NPS (bottom) and its markers separately after correction for possible confounder. (HR: LMR, NLR, chol, alb: high vs. low; NPS: per 1 increase).

In univariable analysis, a significant association could also be found for NLR (high vs. low; HR: 2.271, 95% CI: 1.225–4.211, *p* = 0.009). No associations could be found for serum albumin, total cholesterol or LMR. In multivariable analysis adjusted for TNM-stage, smoking status, and HPV, the strong association of NLR with OS prevailed (HR: 2.558, 95% CI: 1.329–4.922, *p* = 0.005). NPS, serum albumin, total cholesterol and LMR showed no associations with OS or DFS in multivariable analysis. Hazard ratios and 95% CI of the NPS and its included markers for OS are summarized in a Forrest plot in Figure 4.

## 4. Discussion

In this study, we retrospectively applied the Naples Prognostic Score (NPS) to a HNSCC patient cohort and analyzed its association with OS and DFS. The NPS did not show prognostic significance in HNSCC. Out of four markers included in the NPS, only one showed an association with disease outcome in sensitivity analysis. These findings suggest that the NPS may not be universally applicable across tumor entities and highlight the possible need for tumor-specific validation of composite prognostic scores.

Staging of neoplastic disease is essential to guide treatment decision and provide a prognosis on disease outcome. The routinely used system for staging of HNSCC is the anatomically based TNM staging system. However, it does not account for host-related factors such as systemic inflammation and nutritional status, which have been shown to influence disease progression and treatment response [17,18,19,20]. Additional markers could potentially improve the prognostic accuracy of disease staging and help identify patients with high risk of disease recurrence or treatment failure.

Among various blood-based biomarkers derived from routine laboratory parameters, several have demonstrated independent prognostic value in solid tumors [21,22,23,24]. To further increase the prognostic accuracy, composite scores like the prognostic nutritional index (PNI), the systemic immune-inflammation index (SII) or the advanced lung cancer inflammation index (ALI) have been proposed and evaluated [20,25,26,27]. However, many of these scores lack standardized cutoff values, limiting their generalizability and clinical applicability [28,29,30]. The NPS represents a novel score that incorporates multiple parameters and provides a defined scoring system. It has been first evaluated in colorectal cancer and has shown a high prognostic power as reported by Galizia et al. [8].

Since then, it has been validated in various other solid tumors [9,10,11,12]. In this study, however, the NPS did not show significant prognostic power in HNSCC. This discrepancy may reflect tumor-specific differences in metabolic pathways and nutritional status, as well as HPV-associated tumor biology, which could limit the applicability of the NPS in HNSCC. In particular, factors such as HPV status, and tumor-related impairment of nutritional status, for example due to dysphagia, may differ substantially from other solid tumors [31].

Only one out of four included markers of the NPS showed a significant association with disease outcome, namely the NLR. The NLR showed an independent and highly significant association with disease outcome. This finding corroborates previous work on the prognostic value of NLR in HNSCC [32,33]. Although the exact mechanisms underlying the association between high NLR and worse prognosis of cancer patients remain elusive in detail, it has been shown that neutrophilia inhibits the cytolytic activation of lymphocytes [34]. Increasing numbers of tumor infiltrating lymphocytes, in turn, are associated with better prognosis in cancer patients [35,36]. This finding raises the question whether simple, biologically meaningful markers such as NLR may outperform more complex composite scores in certain tumor types. This could make NLR particularly attractive for clinical risk stratification due to its simplicity and availability.

For all other markers included in the NPS, previous research is not as concise.

The prognostic value of the LMR in HNSCC remains controversial. LMR has failed to show an association with disease outcome in HNSCC in a recent study [37]. In contrast, a meta-analysis from 2018 including seven studies found a significant improved OS with elevated LMR [38]. However, most included studies used data-driven cutoff values without external validation, raising concerns about overfitting and limiting generalizability. In line with this, sensitivity analysis in our cohort did not demonstrate a significant association between LMR and disease outcome.

Similarly, evidence regarding serum cholesterol levels in HNSCC is sparse, and previous studies have not demonstrated a clear association with prognosis [39], which is consistent with our findings.

As described above, serum albumin is a key component of the NPS. Beyond its role in the score, it plays a central role in cancer-associated metabolic processes, as tumors can utilize plasma proteins and their degradation products, contributing to tumor cachexia [7,40]. In advanced disease stages, systemic inflammation and malnutrition further suppress albumin synthesis, reinforcing its role as a biomarker of nutritional and inflammatory status [7].

Despite this biological relevance, evidence regarding the prognostic value of serum albumin in HNSCC remains limited. While albumin has been associated with postoperative complications, no clear association with survival outcomes has been demonstrated [41]. Although serum albumin is included in composites scores such as the PNI and ALI, it has rarely been investigated as an independent prognostic factor [42,43].

Furthermore, our group has previously shown that the Advanced Lung Cancer Inflammation Index (ALI), including body mass index (BMI), serum albumin and NLR [27], is independently associated with disease outcome in HNSCC [28], a finding that has recently been validated in a larger, multicenter study [43]. In contrast to the NPS, the ALI incorporates BMI instead of total cholesterol, which may better reflect the nutritional status of HNSCC patients and could partly explain its superior prognostic performance [44].

This study has some limitations that need to be discussed. First, the retrospective character of this study might have biased our findings. We tried to minimize this by creating a homogenously treated cohort of HNSCC patients. Second, preoperative serum markers were collected in a timeframe of approximately 3 weeks to one day preoperatively without assessment of local or systemic infection at the time of sample collection. However, since all blood samples were taken in preparation for major cancer surgery, it is unlikely that patients were suffering from severe infection. Third, this study is a single-center study with a medium sized cohort and without external validation. Although a significantly larger cohort might provide the statistical power for the observed numerical differences in OS and DFS to reach significance, more reliable markers like the NLR are available. Altogether, large prospective studies are lacking to validate the clinical significance of those markers. Additionally, other potential risk factors, such as oral health status, which has been associated with the development of head and neck cancer and may also influence survival outcomes, were not available in our dataset and could therefore not be considered in the present analysis [45]. Similarly, data on comorbidities, which may also affect survival, were not available [46]. Data on treatment selection criteria and performance status were also not collected, which may have influenced survival outcomes. Furthermore, the cohort is heterogenous regarding tumor site, HPV status, and treatment modalities, which may further affect the interpretation and generalizability of our findings. These limitations may have influenced the observed lack of prognostic significance of the NPS and should be considered when interpreting the result.

## 5. Conclusions

The preoperative Naples Prognostic Score showed no significant association with disease outcome in surgically treated HNSCC in this study. A sensitivity analysis for all included markers revealed the NLR to be the only component significantly associated with survival, making it feasible to utilize NLR as an easily obtainable prognostic marker.

## Figures and Tables

**Table 1:** Baseline characteristics of patients included in the study.

	*n* = 140
Gender	
Female	30 (21%)
Male	110 (79%)
Age at diagnosis	60.6
(Q1–Q3)	(54–65)
T-stage	
T1	31 (22%
T2	78 (56%)
T3	17 (12%)
T4	14 (10%)
N-stage	*n* = 81
N0	22 (27%)
N1	18 (22%)
N2a	6 (7%)
N2b	26 (32%)
N2c	8 (10%)
N3	1 (1%)
	*n* = 55
pN0 (HPV+)	5 (9%)
pN1 (HPV+)	25 (45%)
pN2 (HPV+)	23 (42%)
pN3 (HPV+)	2 (4%)
TNM-Staging	
I	23 (16.4%)
II	42 (30%)
III	21 (15%)
IVA	53 (37.9%)
IVB	1 (0.7%)
HPV	*n* = 136
−	81 (60%)
+	55 (40%)
Primum	
Hypopharynx	18 (13%)
Larynx	12 (9%)
Oral Cavity	30 (21%)
Oropharynx	80 (57%)
Alcohol consumption	*n* = 125
Non-drinker	83 (66%)
Active drinker	42 (34%)
Smoking status	*n* = 135
Non/Ex-Smoker	58 (43%)
Smoker	77 (57%)
Therapy	
surgery	8 (6%)
+radiotherapy	108 (77%)
+radiochemotherapy	24 (17%)

**Table 2:** Preoperative Naples Prognostic Score: Uni- and multivariable time-to-event analysis.

	Univariable			Multivariable		
	HR	95% CI	*p*-Value	HR	95% CI	*p*-Value
**Overall survival**						
Naples score group 1 vs. 0	1.809	0.543–6.029	0.334	1.529	0.449–5.201	0.496
Naples score group 2 vs. 0	2.490	0.711–8.713	0.153	1.897	0.531–6.768	0.324
albumin low vs. high	1.525	0.800–2.905	0.199	1.241	0.625–2.463	0.537
cholesterol low vs. high	1.014	0.528–1.946	0.966	1.069	0.540–2.114	0.848
**NLR high vs. low**	**2.271**	**1.225–4.211**	**0.009**	**2.558**	**1.329–4.922**	**0.005**
LMR low vs. High	0.952	0.454–1.955	0.897	0.754	0.350–1.623	0.471
**Disease-free survival**						
Naples score group 1 vs. 0	0.960	0.398–2.315	0.929	0.805	0.327–1.980	0.638
Naples score group 2 vs. 0	1.546	0.612–3.901	0.356	1.209	0.466–3.139	0.695
albumin low vs. high	1.218	0.679–2.186	0.507	1.112	0.607–2.038	0.729
cholesterol low vs. high	1.018	0.574–1.805	0.950	1.108	0.610–2.012	0.736
**NLR high vs. low**	**2.464**	**1.425–4-259**	**0.001**	**2.439**	**1.382–4.306**	**0.002**
LMR low vs. High	0.878	0.460–1.674	0.693	0.691	0.354–1.342	0.278

## Data Availability

The data that support the findings of this study were derived from a retrospectively collected, anonymized patient database at the Medical University of Vienna, Vienna, Austria. These data are not publicly available due to the privacy and ethical restrictions. Aggregated data supporting the results reported in this study are available from the corresponding author upon request.

## Abbreviations

The following abbreviations are used in this manuscript:

AJCC	American Joint Commission on Cancer
ALI	advanced lung cancer inflammation index
BMI	body mass index
CI	confidence interval
DFS	disease-free survival
HR	hazard ratio
LMR	lymphocyte-to-monocyte ratio
NLR	neutrophil-to-lymphocyte ratio
NPS	Naples Prognostic Score
OPSCC	oropharyngeal squamous cell carcinoma
OS	overall survival
PNI	prognostic nutritional index
SII	systematic immune-inflammation index
